# Porous Mg–Zn–Ca scaffolds for bone repair: a study on microstructure, mechanical properties and in vitro degradation behavior

**DOI:** 10.1007/s10856-023-06754-y

**Published:** 2024-03-25

**Authors:** Lei Huo, Qiang Li, Linlin Jiang, Huiqin Jiang, Jianping Zhao, Kangjian Yang, Qiangsheng Dong, Yi Shao, Chenglin Chu, Feng Xue, Jing Bai

**Affiliations:** 1 Taixing Second People’s Hospital, Taizhou, 225411 China; 2 Jiangsu Key Laboratory for Advanced Metallic Materials, School of Materials Science and Engineering, Southeast University, Nanjing, 211189 China; 3 Jiangsu Key Laboratory of Advanced Structural Materials and Application Technology, School of Materials Science and Engineering, Nanjing Institute of Technology, Nanjing, 211167 China; 4 Institute of Medical Devices (Suzhou), Southeast University, Suzhou, 215000 China; 5 Jiangsu Key Laboratory for Light Metal Alloys, Nanjing Yunhai Special Metals Co., Ltd., Nanjing, 211200 China

**Keywords:** Bone tissue engineering, Porous Mg-based scaffolds, Mechanical property, Degradability

## Abstract

Biodegradable porous Mg scaffolds are a promising approach to bone repair. In this work, 3D-spherical porous Mg–1.5Zn–0.2Ca (wt.%) scaffolds were prepared by vacuum infiltration casting technology, and MgF_2_ and fluorapatite coatings were designed to control the degradation behavior of Mg-based scaffolds. The results showed that the pores in Mg-based scaffolds were composed of the main spherical pores (450–600 μm) and interconnected pores (150–200 μm), and the porosity was up to 74.97%. Mg-based porous scaffolds exhibited sufficient mechanical properties with a compressive yield strength of about 4.04 MPa and elastic modulus of appropriately 0.23 GPa. Besides, both MgF_2_ coating and fluorapatite coating could effectively improve the corrosion resistance of porous Mg-based scaffolds. In conclusion, this research would provide data support and theoretical guidance for the application of biodegradable porous Mg-based scaffolds in bone tissue engineering.

**Graphical Abstract:**

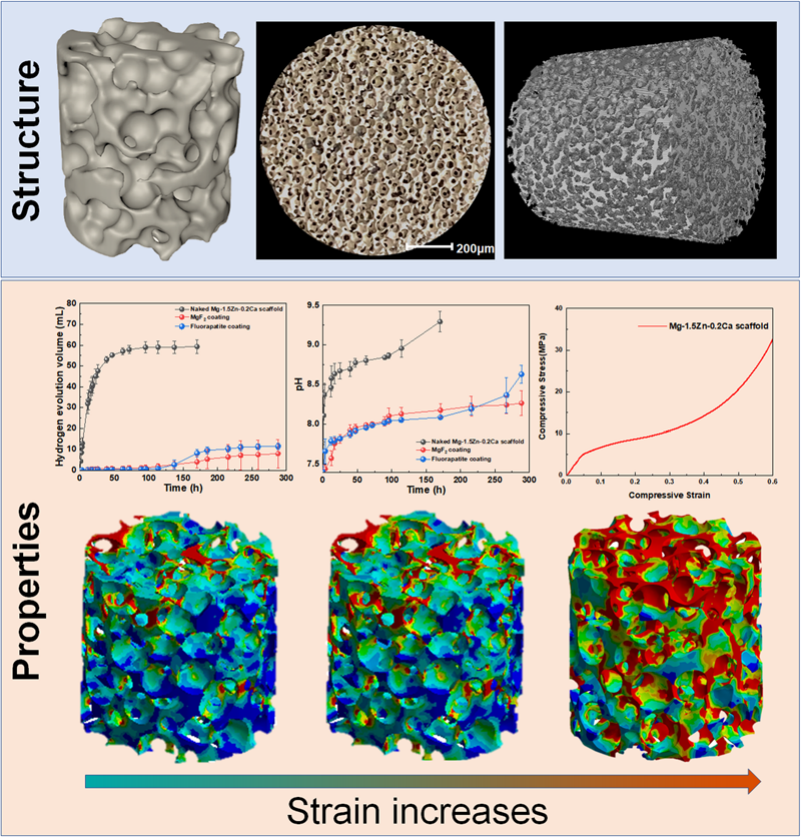

## Introduction

Tissue engineering scaffolds have been developed as a promising strategy for the regeneration of critical-sized bone defects [1–3]. Biomaterials for bone tissue engineering have been supposed as interconnected open-porous structures for cell growth and nutrient transport [4]. In addition, to provide structure support for the ingrowth of new tissue, the scaffold should meet certain mechanical properties. Moreover, bone tissue engineering scaffolds require biocompatibility and biodegradability in terms of biological requirements. Up to now, many biodegradable biomaterials have been developed as tissue engineering scaffolds, such as synthetic polymer materials(e.g. PLLA [5] and PCL [6, 7]), bioactive ceramic materials [8], and biodegradable metals (e.g. Mg [9], Zn [10], Fe [11]).

Mg and its alloys have been paid much attention owing to their suitable mechanical properties (such as elastic modulus and density close to human bone [12]), favorable biodegradability [13, 14], and good biocompatibility [15]. Besides, Mg is one of the macro elements in the human body, playing an osteoinductive and osteoconductive role in the growth of bone tissue and the healing of bone fractures [16]. Thus, based on the overall consideration of mechanical properties, biodegradation, and biocompatibility, Mg-based scaffolds are expected to be a new generation of tissue engineering scaffolds.

Recently, many studies have been carried out to evaluate the effects of the preparation process and pore structure regulation of porous Mg-based scaffolds on the chemical-physical properties [17–19]. The preparation methods of Mg scaffolds include vacuum infiltration casting [17], powder metallurgy [20], additive manufacturing [21, 22], laser perforation [23], and so on. Herein, in vacuum infiltration casting technology, the pore size and porosity of Mg-based scaffolds are relatively controllable [18]. Mg-based scaffolds with spherical pores exhibited better resistance to the deterioration of the interconnectivity and higher porosity compared with irregular pores [19]. However, Mg scaffolds exhibited enhanced biodegradation rate owing to their chemical nature and large exposed surface from pore structure. Therefore, surface modifications should be introduced to regulate the degradation rate. Yu et al. [23] reported that MgF_2_ coatings simultaneously enhanced the corrosion resistance, biocompatibility, osteoconductivity and osteoinductivity of Mg-based scaffolds.

Based above, this work investigated Mg scaffolds with spherical pore structures and introduced surface coatings for controlling the degradation rate. The pore structure, mechanical properties, and in vitro degradability of porous Mg-based scaffolds were comprehensively evaluated.

## Materials and methods

### Materials

**Fig. 1:**
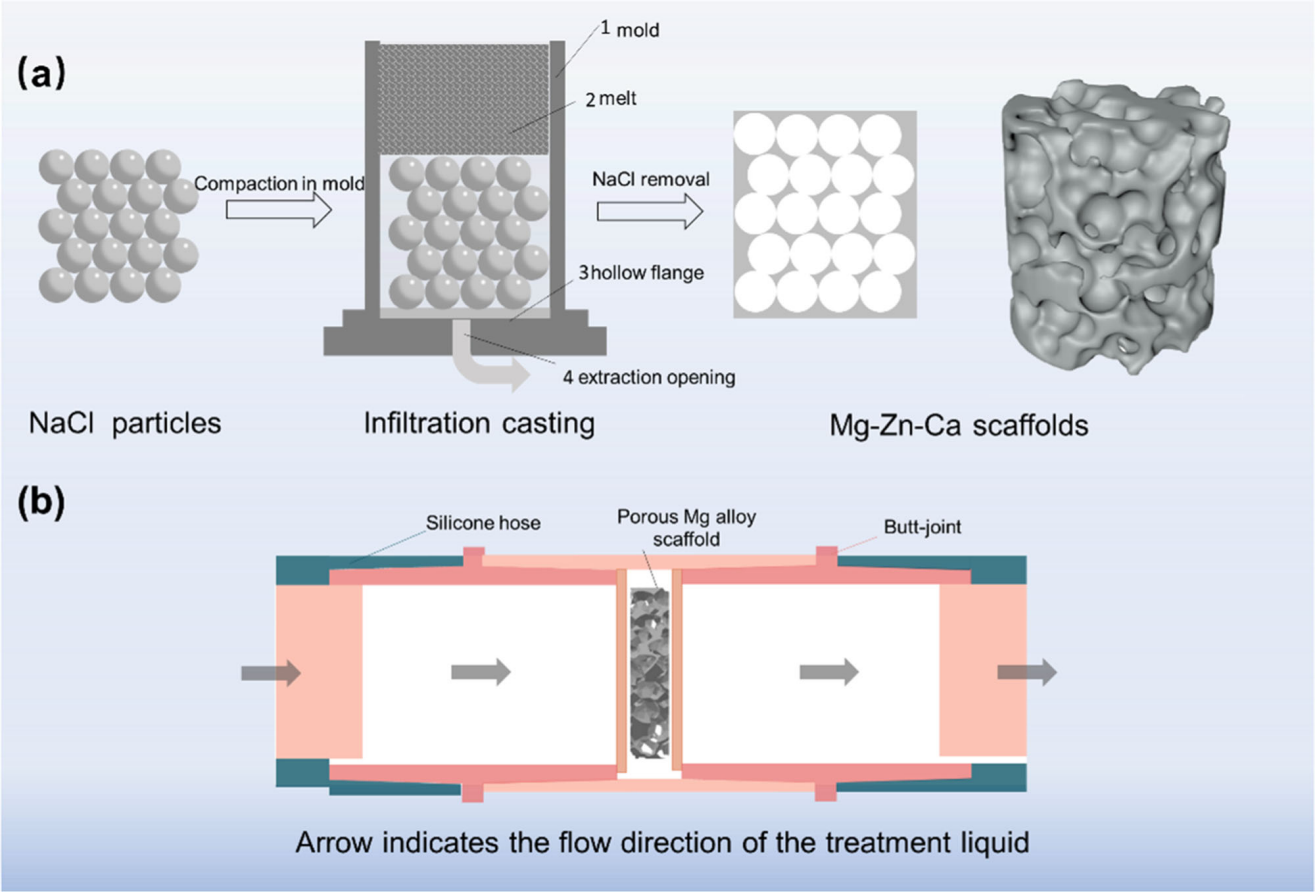
**a** Schematic diagram of preparation process of Mg-based scaffolds; **b** flowing device for preparing calcium phosphate coating

In this study, the raw materials for Mg–Zn–Ca alloy were pure Mg (≥99.95 wt%), pure Zn (≥99.95 wt%), and Mg-30wt.% Ca alloy. Mg–1.5Zn–0.2Ca (wt.%) scaffolds were prepared by vacuum infiltration casting. The pore former for preparation of porous materials was spherical NaCl particles with a mean size of 450–600 μm. The schematic diagram of preparation, including NaCl pretreatment, infiltration casting of Mg-based scaffolds, and NaCl removal, is shown in Fig. 1a.

### Scaffolds

Firstly, NaCl templates were put into a casting mold (Ф 60 mm × 260 mm) and heated to 400 °C. Secondly, the mold containing the NaCl templates was heated to 660 °C under a mixed protective gas of CO_2_ and SF_6_. Thirdly, Mg ingots were cleaned with NaOH solution (approximately 1 mol/L) to remove NaCl templates.

### Characterizations

The surface morphologies were observed by scanning electron microscopy (SEM, Sirion 200) with an energy dispersive X-ray spectrometer (EDS, Oxford). The phase composition was analyzed by an X-ray diffractometer (XRD, Bruker D8-Discover) with a scan angle of 20°–90°. The 3D model of the porous Mg-based scaffold was collected and reconstructed by computerized tomography (CT, CT, YXLON CT Precision). The pore size distribution in the as-prepared scaffolds was collected by the Image-Pro Plus 6.0 program and the porosity was calculated by VGSTUDIO MAX software.

### Mechanical properties

The compression performance was measured by an electronic universal testing machine (SANS CMT4503). Cylindrical samples with a dimension of Ф 10 mm × 10 mm were used for the evaluation of mechanical properties. The compression tests were carried out at a compression speed of 0.5 mm/min at room temperature, and repeated at least three times in each case.

### Simulation analysis

Digital image correlation technology (DIC) was utilized to achieve the real strain distribution of porous Mg-based scaffolds. Finite element analysis (FEA) was applied to simulate the strain distribution of Mg-based scaffolds under compressive loading. The model for FEA was selected from CT analysis to reliably simulate the mechanical behavior of Mg scaffolds. The meshes were divided into triangular elements with a size of 0.1 mm. The main parameters of the material were set (0.23 GPa for elastic modulus, 0.3 for Poisson’s ratio, and 4.0 MPa for compressive yield strength, respectively). Herein, the ultimate compression distance was 1.2 mm to ensure that the material reached the plastic deformation stage.

### Scaffold degradation

Hanks’ solution (NaCl (8.00 g/L), KCl (0.40 g/L), CaCl_2_ (0.14 g/L), NaHCO_3_ (0.35 g/L), MgCl_2_·6H_2_O (0.10 g/L), MgSO_4_·7H_2_O (0.06 g/L), Na_2_HPO_4_ (0.06 g/L), KH_2_PO_4_ (0.06 g/L) and C_6_H_12_O_6_ (1.00 g/L) [24, 25]) was used for evaluation of in vitro degradation behaviors. The specimens for corrosion tests were cut into a dimension of Ф 10 mm × 2 mm. The degradation behavior was evaluated in Hank’s solution at 37 ± 0.5 ^o^C, and the degradation performance was comprehensively characterized in terms of the hydrogen evolution curve, pH curve, and the change of surface morphology before and after degradation. After immersion, the corrosion products were removed in a solution containing 200 g/L CrO_3_ and 10 g/L AgNO_3_. The fluorination treatment was performed by immersing the Mg-based scaffolds in a poly tetra fluoroethylene reaction vessel filled with 40 wt.% HF solution, immersed at 80 °C for 40 h. The calcium phosphate coating was designed to grow on the surface of the MgF_2_ coating. The porous Mg-based scaffolds were immersed for 2 h in the flowing treatment solution containing 0.06 mol/L NH_4_H_2_PO_4_, 0.1 mol/L Ca(NO_3_)_2_·4H_2_O, as shown in Fig. 1b.

## Results and discussion

### Structure characterization

**Fig. 2:**
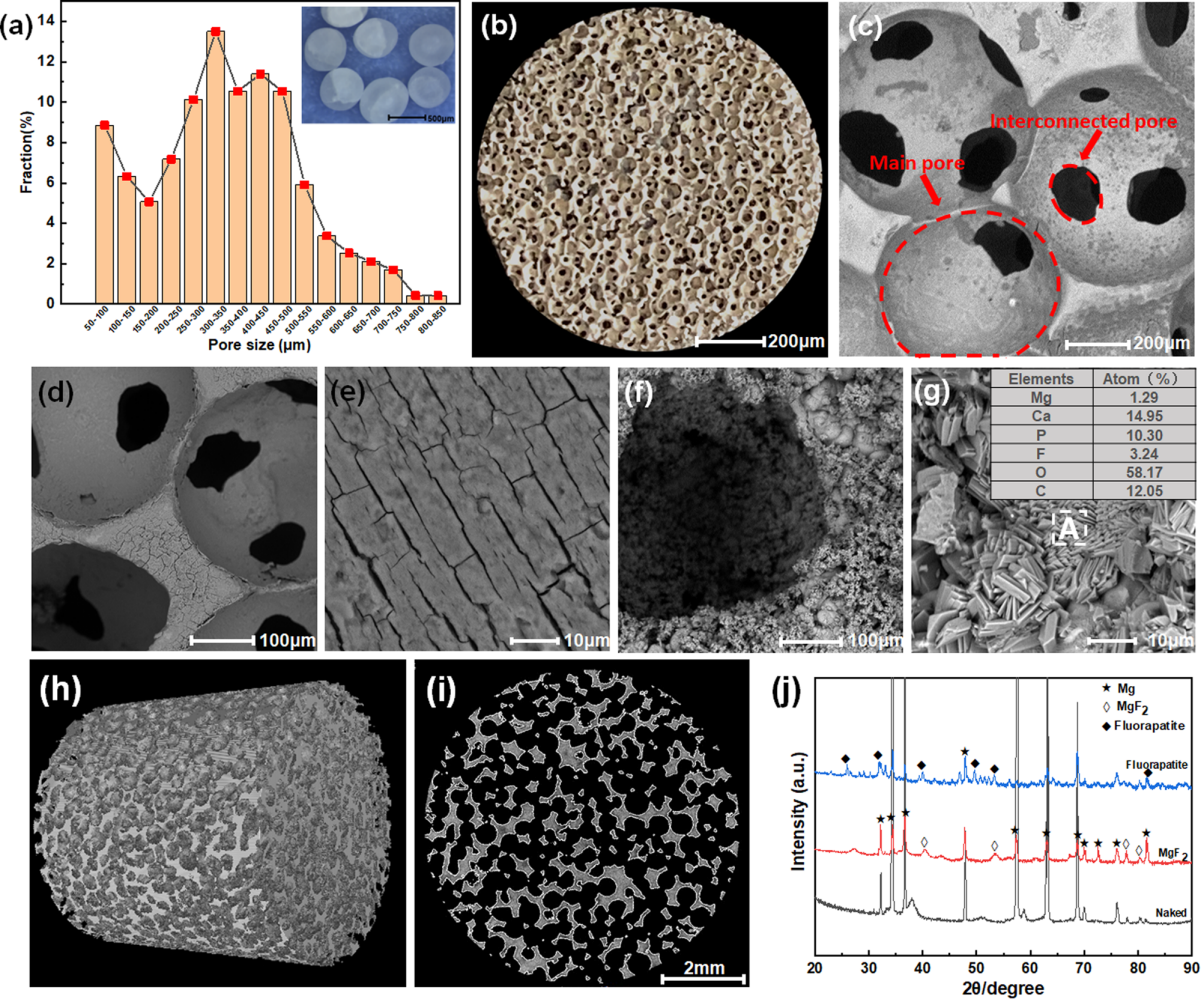
Pore structure characterizations of as-prepared porous Mg-based scaffolds and microstructure analysis: (**a**) the morphologies of NaCl templates and pore size distribution in Mg-based scaffolds, **b** OM, **c** SEM, **d**, **e** the morphologies of as-prepared porous Mg-based scaffolds with MgF_2_ coating, **f**, **g** the morphologies of as-prepared porous Mg-based scaffolds with fluorapatite coating, **h** CT 3D model, **i** CT cross-section morphology, **j** XRD patterns

As shown in Fig. 2a, NaCl templates are typical 3D spheres of Ф 450–600 μm. The pore size of Mg-based scaffolds mainly ranges from 50 to 800 μm, which was collected by Image-Pro Plus 6.0 program. Figure 2b, c shows pore structure characterizations of porous Mg-based scaffolds. The open-pore structures (in Fig. 2b) are composed of two main pores, including main pores (450–600 μm) and interconnected pores (150–200 μm), as displayed in Fig. 2a, c. Figure 2d, e shows the micromorphology of porous Mg-based scaffolds with MgF_2_ coating. The coating on the outer surface is relatively flat with directional strip microcracks. Figure 2f, g shows the morphologies of fluorapatite coating. It can be observed that the fine particles are composed of needle-like clusters of 3–6 μm, and the inclusions are distributed with rectangular thick lamellar crystal clusters shown in Fig. 2g. Besides, X-ray diffuse peaks of MgF_2_ and fluorapatite phases were detected on Mg-based scaffolds with MgF_2_ and fluorapatite coatings, respectively, as shown in Fig. 2j.

The 3D model of the as-prepared porous Mg-based scaffolds was reconstructed in Fig. 2h by CT. Figure 2i is the cross-section view of Mg-based scaffolds from Fig. 2h. It displays that the pores are uniformly distributed with suitable connectivity between the pores. Herein, the central area (Ф 8 mm × 8 mm) is selected to calculate pore structural parameters. The measured porosity is 74.97%, close to 75%, and the specific surface area is 8.36 mm^2^/mm^3^.

### Mechanical properties

**Fig. 3:**
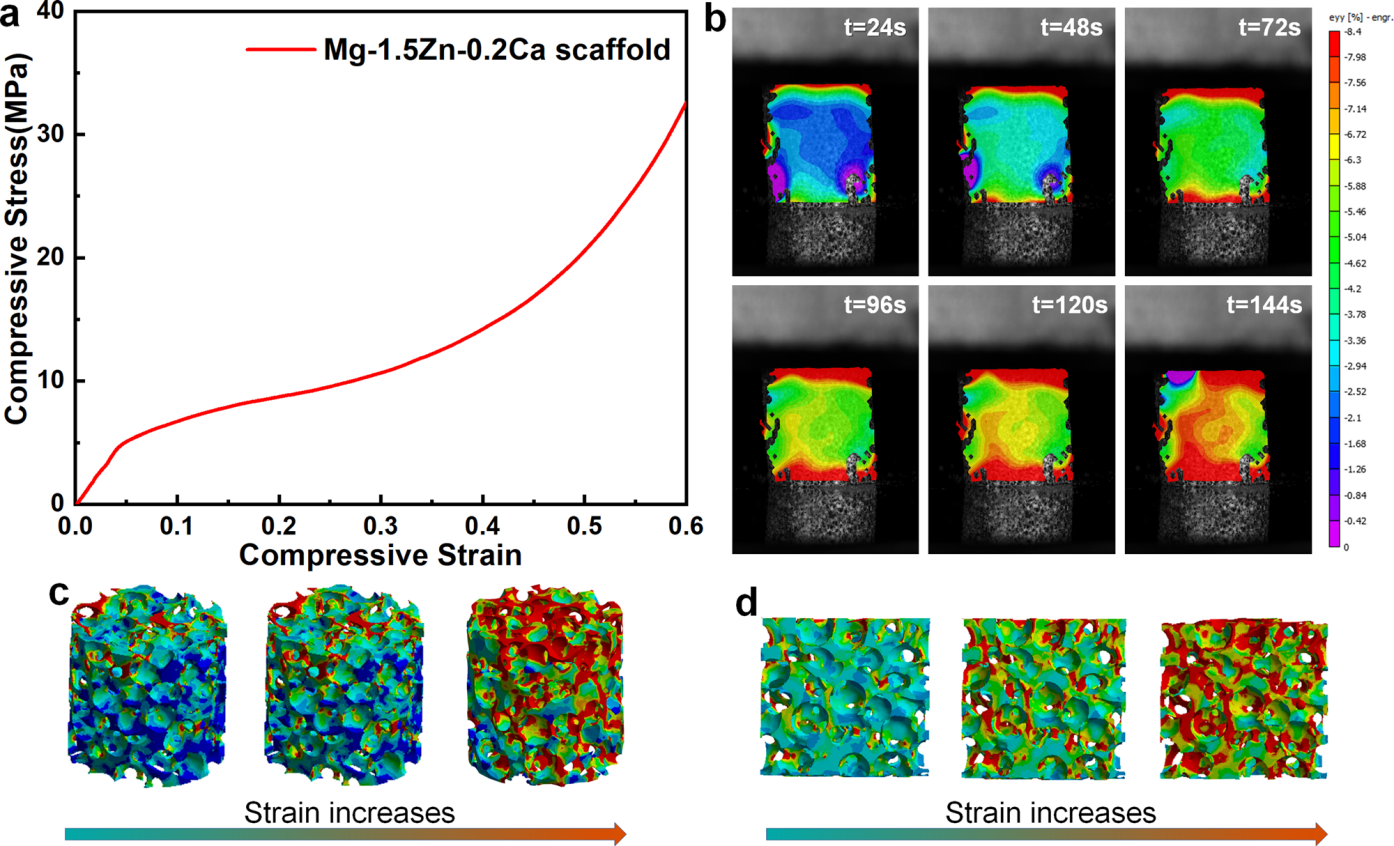
Compressive stress-strain curve (**a**), Strain distribution process (**b**), strain distribution (**c**) and its longitudinal section (**d**) under compressive loading by FE modeling

Figure 3 shows the mechanical properties of as-prepared Mg-based scaffolds. Figure 3a exhibits the strain distribution process of porous Mg-based scaffolds. Figure 3b, c displays compressive strain distribution obtained by FE modeling. During the entire compression process, the maximal strain is distributed at both ends of the specimens, while the lower strain is inside.

A typical compression stress-strain curve is illustrated in Fig. 3d, which consists of the elastic stage, yield stage and densification stage [17]. During the elastic stage, the inhomogeneity of the porous structure leads to local stress concentration under the lower strain. The weakest part of the pore walls undergoes bending, stretching, and compression. The pore walls are not bent and fractured until the local stress exceeds the yield stress of the material, which leads to early local plastic instability. However, the curve shows roughly linear changes due to the stable pore structure. With the further increase of the compressive load, most pore walls are buckled and collapsed, which is corresponding to the yield stage. In the densification stage, the compressive stress continues to increase so that the porous structure is collapsed completely. Meanwhile, the slope of the stress-strain curve increased rapidly. According to Fig. 3d, the compressive yield strength and compressive elastic modulus of the porous Mg-based scaffolds are 4.04 ± 0.85 MPa and 0.23 ± 0.12 GPa, respectively. Table 1 summarizes the characterizations of porous Mg scaffolds. Alloying and pore structure are both important factors in mechanical properties [17]. Owing to alloying strengthening, Mg-Zn-Ca scaffold in the present work exhibited higher mechanical properties than Mg scaffold reported by Jia et al. [18]. In this present work, the porous Mg-based scaffolds showed advantages in the comprehensive evaluation of porosity and mechanical properties, which are matched with human cancellous bone [26].

**Table 1:** Summary of relevant research on porous Mg scaffolds

Alloys	Pore size (μm)	Porosity (%)	Yield strength (MPa)	Young’s modulus (GPa)	Reference
Mg	/	64.4–76.9	20.05–13.18	/	[30]
Mg–xZn–0.3Ca	400–500	73.4 ± 2.5	5.2 ± 0.3	0.39 ± 0.05	[17]
Mg	200–350	75.14 ± 0.35	0.86 ± 0.05	0.12 ± 0.02	[18]
Mg–Nd–Zn–Zr	400–450	70.58 ± 0.91	6.87 ± 0.16	0.40 ± 0.09	[31]
Mg–4Li–4Al–2RE	400–500	65	/	/	[32]
Mg–2Y–1Zn–1Mn	500–600	55	2.06 ± 0.78	0.068 ± 0.028	[33]
Mg–1.5Zn–0.2Ca	450–600	74.97	4.04 ± 0.85	0.23 ± 0.12	Present work

### Degradation behavior

Degradation of Mg-based alloys is always accompanied by hydrogen evolution, as shown in Eq. (1) [27, 28].

1 $${\rm{Mg}}+2{{\rm{H}}}_{2}{\rm{O}}={{\rm{Mg}}}^{2+}+2{{\rm{OH}}}^{-}+{{\rm{H}}}_{2}$$

**Fig. 4:**
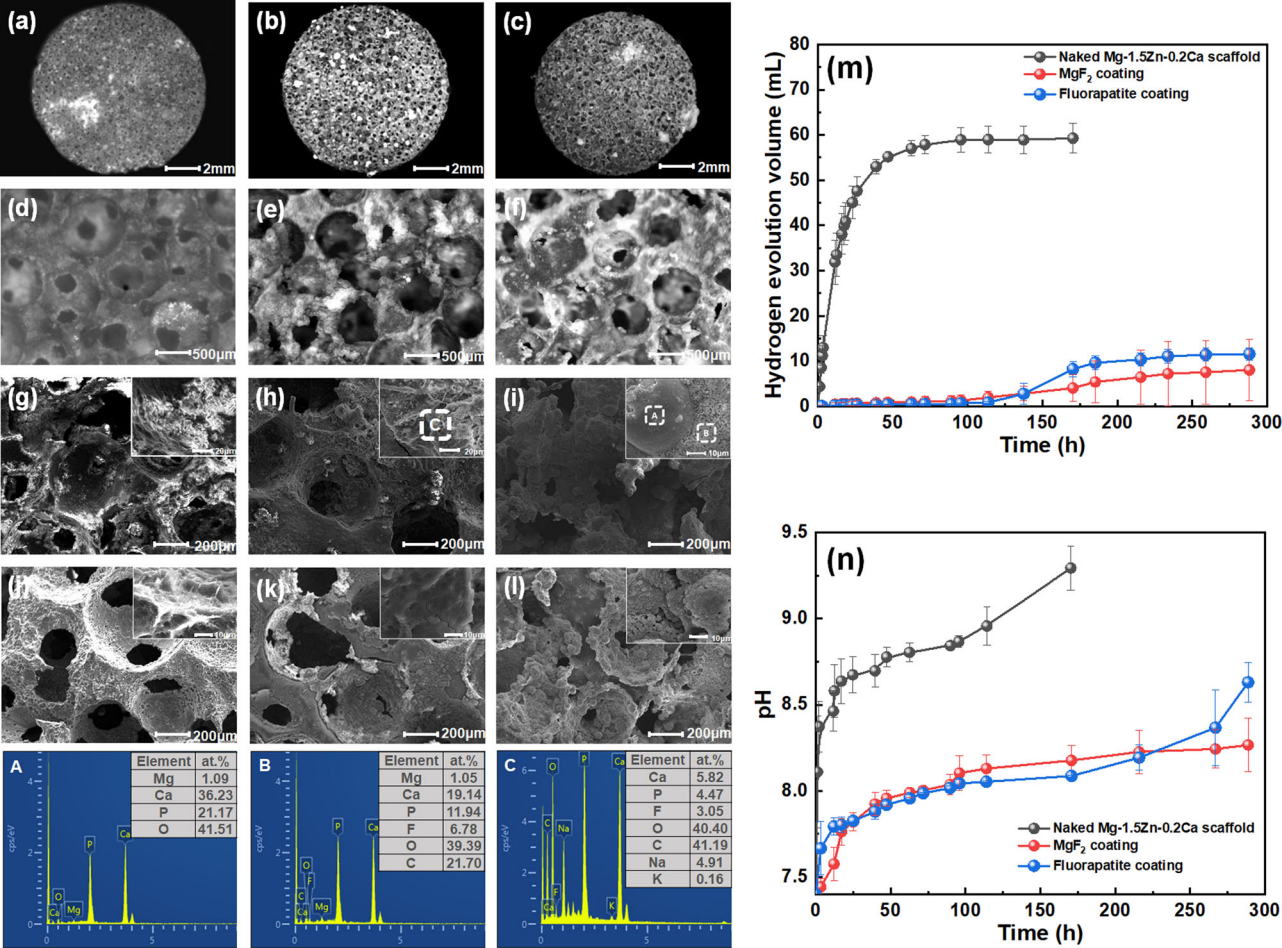
Degradation test results: **a**–**f** macroscopic morphologies of naked, MgF_2_- and fluorapatite- coated porous scaffolds immersed in Hanks’ solution for 300 h, **g**–**i** microscopic corrosion morphologies and EDS elements analysis (A, B and C), **j**–**l** de-corrosion morphologies, **m** hydrogen evolution curve, **n** the pH change curve (**a**, **d**, **g**, **j**: naked Mg scaffold, **b**, **e**, **h**, **k**: MgF_2_-coated Mg scaffold, **c**, **f**, **i**, **l**: fluorapatite-coated Mg scaffold)

Figure 4 shows the degradation test results. Figure 4m, n displays the hydrogen evolution curves and pH curves, and the hydrogen evolution rate is consistent with pH. Mg-based scaffolds with MgF_2_ coating and fluorapatite coating both exhibit a lower degradation rate than the uncoated Mg-based scaffolds, while the corrosion rate of coated Mg-based scaffolds increases in the later stage. In summary, the anti-corrosion properties of the coated Mg-based scaffolds are significantly improved.

Figure 4a–c are the macroscopic corrosion morphologies of three porous Mg-based scaffolds immersed in Hanks’ solution for 300 h. The 3D-spherical porous structure characteristics remain intact, but MgF_2_ and fluorapatite are covered on the surface. Herein, fluorapatite, as a representative bioceramic, exhibited enhanced bio bioactivity [29]. Figure 4g–i reveal the corrosion morphologies and EDS results. For fluorapatite coating, there are more calcium-phosphorus products covering on the scaffold, as shown in EDS analysis of area A and B. The pores are gradually covered by corrosion products, protecting the porous Mg alloy substrate from further corrosion. The calcium-phosphorus compounds in area A has grown massively on the surface of the fluorapatite coating, which is corresponding to fewer Mg elements. Figure 4j–l are the morphologies of porous Mg-based scaffolds immersed for 300 h after removing corrosion products. The porous structure characteristics of three Mg-based scaffolds remain intact. However, the increase in biodegradation rate at the late immersion stage is ascribed to cracks growth (in Fig. 4h). There are a small number of corrosion pits and thick needle-like clusters observed on the surface of the fluorapatite film, as shown in Fig. 4i. Although the fluorapatite coating exhibits local failure, the majority of the coating still perfectly adheres to the porous Mg substrate. Both MgF_2_ and fluorapatite enhance the corrosion resistance of Mg-based scaffolds.

## Conclusions

In this work, 3D-spherical Mg-based scaffolds for bone repair were prepared with an average pore size of 450–600 µm and a porosity of 74.97%. The Mg-based scaffolds exhibited a compressive yield strength of 4.04 MPa and a compressive elastic modulus of 0.23 GPa. In addition, the real and simulated strain distributions were in good agreement. The Mg-based scaffolds meet the mechanical requirements for cancellous bone repair. In terms of degradation behavior, both MgF_2_ coating and fluorapatite coating effectively improve the corrosion resistance of Mg-based scaffolds. The pore structure of the scaffolds remains intact with tiny corrosion products when immersed in Hanks’ solution for 300 h. The fluorapatite coating provided better protection for Mg-based scaffolds. Biodegradable Mg-based scaffolds for bone tissue engineering are still in their infancy. The related alloying elements, preparation process, and surface coating need further research to control the degradation rate of Mg. Furthermore, combining Mg scaffolds with biopolymers, bioactive ceramics, and drugs will be a direction of future development.
